# A pilot trial of the peer-based distribution of HIV self-test kits among fishermen in Bulisa, Uganda

**DOI:** 10.1371/journal.pone.0208191

**Published:** 2018-11-29

**Authors:** Augustine T. Choko, Mastula Nanfuka, Josephine Birungi, Geoffrey Taasi, Prossy Kisembo, Stephane Helleringer

**Affiliations:** 1 Malawi- Liverpool Wellcome Trust Clinical Research Programme, Blantyre, Malawi; 2 Dept. of Infectious Disease Epidemiology, London School of Hygiene & Tropical Medicine, London, United Kingdom; 3 The AIDS Support Organisation (TASO), Kampala, Uganda; 4 Ministry of Health, Kampala, Uganda; 5 John Hopkins University, Baltimore, Maryland, United States of America; Boston University School of Public Health, UNITED STATES

## Abstract

**Background:**

HIV self-testing (HIVST) addresses barriers to HIV diagnosis among men, but current approaches to distributing HIVST kits only reach a subset of the men requiring testing.

**Methods:**

We conducted a pilot trial of the secondary distribution of HIVST kits through peer networks in fishing communities of Buliisa district (Uganda). We recruited distributors (“seeds”) among male patients of a health facility, and among community members. Seeds were trained in HIVST and asked to distribute up to five kits to their peers (“recruits”). Recruits were referred to the study using a coupon, and asked to return the HIVST kit (used or unused). The accuracy of HIVST was measured against a confirmatory test conducted by a health worker. We conducted audio computer assisted self-interviews to measure the occurrence of adverse events, and evaluate the potential yield of peer-delivered HIVST. We also assessed how seeds and recruits rated their experience with peer-distributed HIVST.

**Results:**

Nineteen seeds offered an HIVST kit to 116 men, and 95 (81.9%) accepted the offer. No recruit reported coercion, but two seeds experienced hostility from recruits or their family members. The sensitivity of peer-distributed HIVST, as interpreted by recruits, was 100%, and its specificity was 92.8%. Among recruits, 29 had never tested (25.8%), and 42 (44.2%) had tested more than a year ago. Three men living with HIV learned their status through peer-distributed HIVST (yield = 1 new diagnosis per 6.3 seeds). Most recruits (85/88) and seeds (19/19) reported that they would recommend HIVST to their friends and family. All seeds stated that they would accept acting as peer distributors again.

**Conclusions:**

This novel peer-based distribution model of HIVST is safe, and has high uptake. It could help reduce the gender gap in HIV testing in under-served fishing communities in Uganda and elsewhere.

## Introduction

Significant numbers of persons living with HIV remain undiagnosed in African countries [1]. To end the HIV/AIDS epidemic by 2030, new HIV testing strategies must be explored. In particular, strategies that reach men living with HIV (MLWH) are needed [2], since MLWH are less likely to use existing testing services than women [3–5]. Novel HIV testing strategies may contribute to ending the HIV/AIDS epidemic by reaching hard to reach groups that continue to contribute to gaps in HIV status knowledge as well as being drivers of HIV transmission.

One potential strategy is HIV self-testing (HIVST), i.e., an individual performing his/her own HIV test [6–10]. HIVST is accurate, convenient and confidential [6, 11–13]. It may foster repeat testing [7], but strategies to reach undiagnosed MLWH with HIVST remain underdeveloped. Current models include distributing HIVST kits at health facilities, or during home visits by health workers [6]. These approaches may miss MLWH who seldom attend health facilities, or who are absent from home for work-related or other reasons. Thus, alternate distribution mechanisms for HIV self-testing targeting MLWH need to be explored.

Several studies have also investigated the secondary distribution of HIVST kits. In this model, some individuals are given kits to distribute to members of their social networks. Most such studies have mobilized women as distributors of HIVST kits: clients of antenatal care clinics have been asked to distribute HIVST kits to their partner [14] and female sex workers (FSW) have been asked to enroll their male clients [14, 15]. Despite being safe and effective, these approaches only reach a subset of the MLWH who require testing.

We recruited men as distributors (or “seeds”), and asked them to offer HIVST kits to their peers. This approach has been used among men who have sex with men [16], but not in other high-risk groups of men. Potential concerns include an increased incidence of coerced testing [17]. We thus measured the safety, uptake and potential yield of this strategy in several fishing communities in Uganda.

## Methods

We conducted a single-arm pilot trial of the peer-based distribution of HIVST kits in 4 fishing communities of Buliisa district, on the shores of Lake Albert. As in other fishing communities in Uganda [18], HIV risk is high, but HIV testing is limited [11]. The institutional review boards of Johns Hopkins University, the AIDS Support organization and the Uganda National Council for Science and Technology approved the study.

We translated materials detailing HIVST procedures into the local language (Runyoro). We developed standardized scripts (S1 ScriptEng) to help men 1) introduce HIVST to peers, 2) address misunderstandings and 3) troubleshoot conflictual situations that may arise during the distribution of HIVST kits. We then recruited seeds among a) patients receiving antiretroviral treatment at a local facility of the AIDS Support Organization (TASO), b) clients of other services (e.g., HIV testing and counseling) or c) community members. Seeds had to be male, 18 years or older and residents of the study communities. They also had to report fishing as their primary economic activity. All seeds gave written or witnessed consent by thumb print before participating in study procedures.

We used the OraQuick ADVANCE Rapid HIV-1/2 Antibody Test (OraSure Technologies), an oral-fluid based test kit. Each kit was packaged with a leaflet detailing how to conduct HIVST and interpret results. Two study assistants trained the seeds by giving an overview of HIVST procedures, a discussion of recruitment scripts (S1 ScriptEng), and an explanation of the instructional leaflet. They offered seeds up to 5 HIVST kits for distribution and asked them to target their peers above 18 years old who had not recently tested. In turn, the seeds provided training to their peers on how to self-test correctly including a demonstration using the scripts received during seeds’ training. A key component of the seeds training was confidentiality, the peers who ultimately received the self-test kits were neither mandated to self-test in the presence of the seeds nor required to disclose their results to the seeds. These peers would then decide on their own to link to the clinic for confirmatory testing and HIV treatment initiation by presenting a pre-allocated coupon.

Study assessments included a baseline interview with seeds to elicit their socio-demographic characteristics and HIV testing history. Seeds were followed-up one month later, to ask how many individuals they had offered HIVST kits to, how many refused, what were the reasons for refusals, and if they experienced hostility from potential recruits or community members. At that time, seeds were also asked to evaluate their experience with the distribution of HIVST kits to peers, and whether they would act as distributors again if asked.

We used referral coupons to enroll into the study the individuals that accepted a HIVST kit (“recruits”) from a seed. These coupons included enrollment information, and an indication that transportation costs would be reimbursed (≈ 5 USD). Recruits were asked to present at the TASO facility, and to return the used or unused HIVST kit. All recruits who presented to the TASO facility gave written or witnessed consent by thumb print before being enrolled in the study Upon enrollment, recruits were asked to complete a questionnaire about their socio-demographic characteristics, relation with the seed, use of HIVST, interpretation of HIVST results, and prior HIV testing history. They were asked to provide information about their decision to use the peer-delivered HIVST kit (or not), and the context in which they used the HIVST kit. For example, we asked recruits if they used the HIVST kit immediately after receiving it, or later, if they tested at home or elsewhere, and if they trusted the results they obtained. We also asked recruits to rate their experience of HIVST, including stating whether they were satisfied with HIVST and whether they would recommend HIVST to friends and relatives.

Since the results of OraQuick test kits are stable even at sub-optimal heat conditions over long periods of time [19], a health worker independently read the results of the returned HIVST kit again, for quality assurance purposes. Finally, recruits were offered the opportunity to undergo confirmatory HIV testing, using the Uganda HIV testing algorithm. This used a serial algorithm in which recruits were first tested with Determine, and then initial positive results were confirmed through STAT-PAK with Unigold as a tie breaker. Results from this test served as reference in our evaluation of the accuracy of peer-distributed HIVST. MLWH diagnosed during the study were linked to care and treatment services offered by TASO.

We used open data kit (ODK) for data collection. Sensitive questions were asked using audio computer-assisted self-interviewing (ACASI) to limit social desirability bias [20]. The primary outcome was the proportion of HIVST recruits who reported being coerced to undergo HIVST. Assuming that each seed would enroll at least two recruits, and using a one-sided test with *α* = 0.05, the trial was powered to detect an increase in coercion from 2% as in prior studies [7] to 10%. Additional outcomes included seeds’ reports of hostile reactions; the proportion of individuals who accepted to self-test; and the sensitivity/specificity of HIVST according to the recruits’ and the health worker’s readings of test results.

Recruits who reported having received positive test results before HIVST were classified as “already diagnosed”. Recruits who reported that HIVST was the first time they obtained positive results were classified as “newly diagnosed” if confirmatory results were positive, and as “false positives” if confirmatory results were negative. Recruits who obtained negative HIVST results were classified as having learned their HIV-negative status if they had never received such a result, and as having received “a repeat HIV-negative test” otherwise.

In our analysis, we first describe the characteristics of seeds and we report the uptake of peer-distributed HIVST, i.e., the numbers of recruits divided by the number of invitations to participate in HIVST extended by seeds. We list the main reasons for not accepting an HIVST offer, as reported by seeds that experienced these refusals. Second, we describe the characteristics of recruits (e.g., age, educational level), and their relationships with the seeds. Third, we report the context within which recruits used HIVST (e.g., whether they tested at home or elsewhere), and we investigate how recruits evaluated their experience of HIVST (e.g., whether they HIVST difficult to perform). Fourth, we report primary and secondary outcomes of the trial, including the occurrence of adverse events, the accuracy of HIVST as performed by the recruits and the potential yield of peer-distributed HIVST. Finally, we report whether seeds and recruits were satisfied with their experience of peer-distributed HIVST, and if they would recommend HIVST to friends and family. Most of our results are descriptive and based on univariate statistics. In some instances however, we test whether an HIVST outcome differed between recruits of seeds who were patients of ART services and those of other seeds. To do so, we use *χ*^2^ tests of the association between categorical variables. In those tests, standard errors are adjusted for the clustering of recruits within seeds. All analyses were conducted using STATA 14.

## Results

### Characteristics of seeds

**Fig 1:**
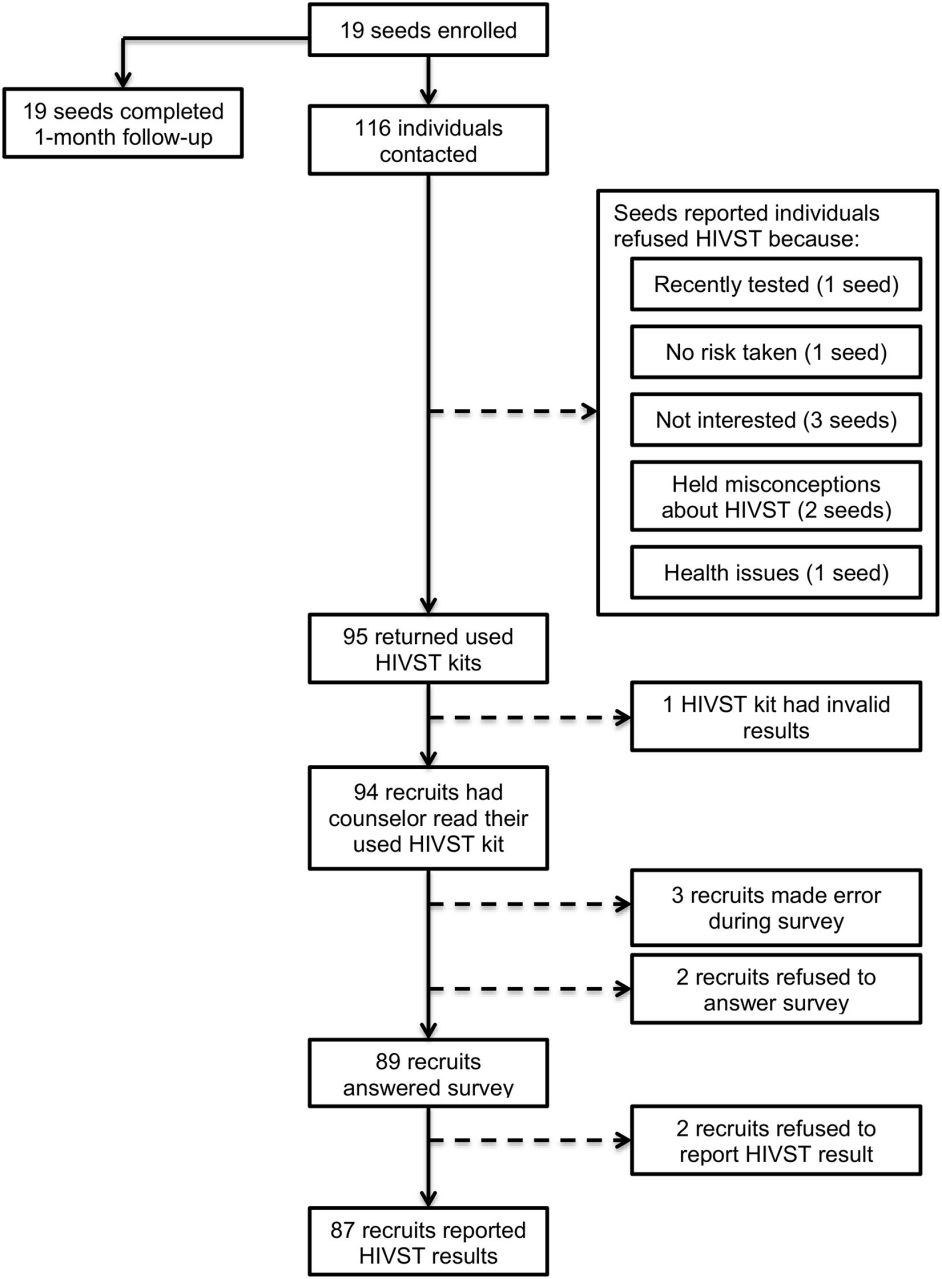
Flow chart of study participation and recruitment.

We enrolled 19 seeds (Fig 1). Among them, 10 were users of ART services, and 9 were users of non-ART services or community members. The youngest seed was 23 years old, whereas the oldest seed was 62 years old. The median age among seeds was 41 years old. The seeds who were recruited through ART services were however older than other seeds: the median age among ART users was 51 years vs. 27 years among other seeds (p<0.001, according to a non-parametric test of the equality of medians). Only one of the seeds (1/19, 5.3%) had never attended primary school, whereas 10 of the seeds had attended secondary school. All seeds reported fishing as their primary economic activity, and only two seeds reported other economic activities (e.g., agriculture).

### Uptake of HIV self-testing

All seeds requested 5 HIVST kits for distribution. They offered HIVST kits to 116 individuals (Fig 1), and 95 accepted the offer (81.9%). Reasons for refusals included among the individuals to whom they offered HIVST kits included having recently tested, not having taken risks, not being interested, holding misconceptions about HIVST and having health issues. Ninety-five recruits returned used HIVST kits, but one kit had invalid results. Eighty-seven recruits reported how they interpreted their HIVST results (91.6%).

### Characteristics of recruits

**Fig 2:**
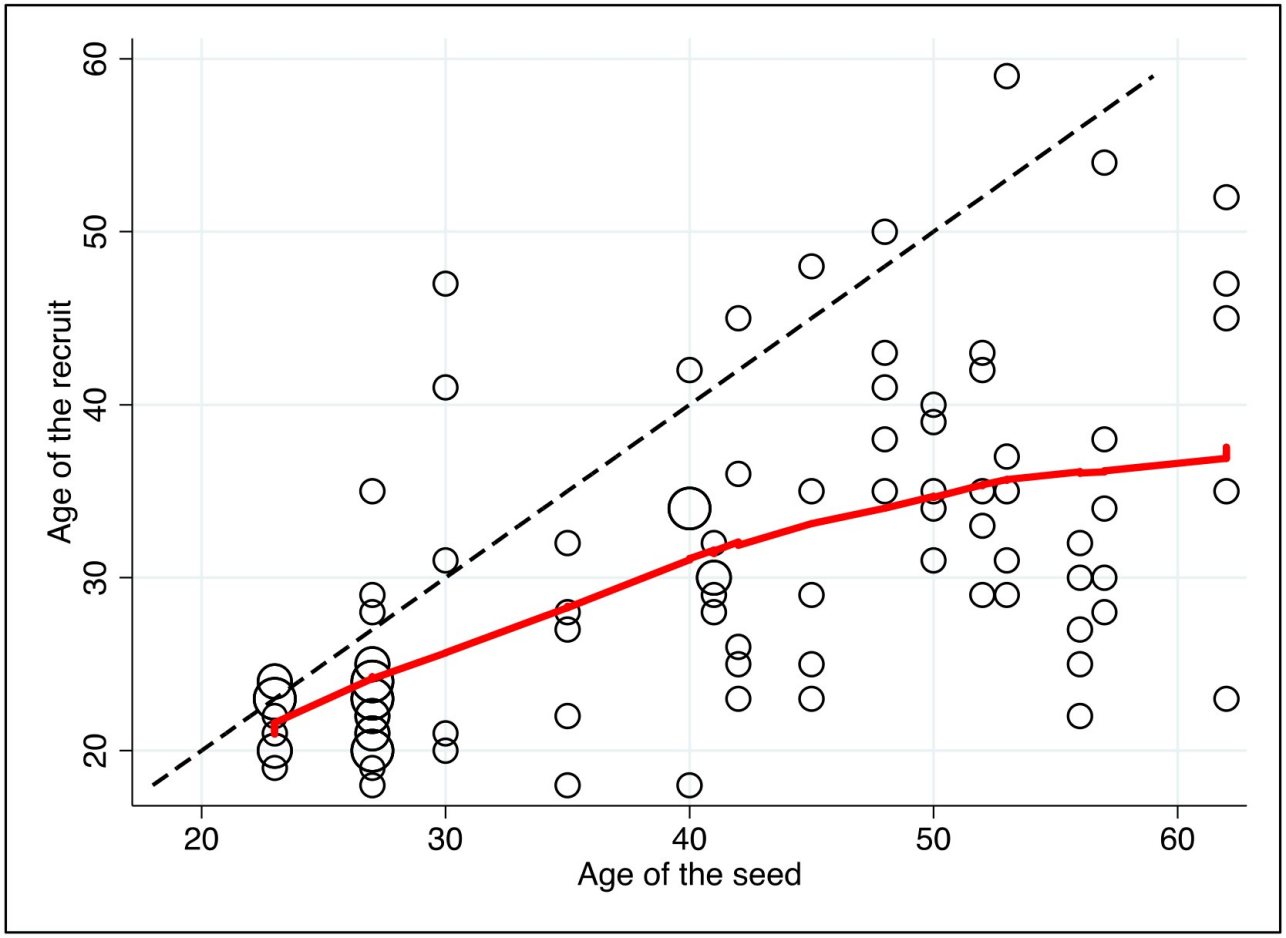
Association between age of the seed and age of the recruits. Notes: each circle represents one or several seed-recruit pairs. The size of the circle is proportional to the number of pairs. The dotted line represents equality between the ages of seeds and ages of recruits. Thus, for dots that lie below that line, the seed was older than the recruit. The red line represents the Lowess fit to the cloud of points.

**Fig 3:**
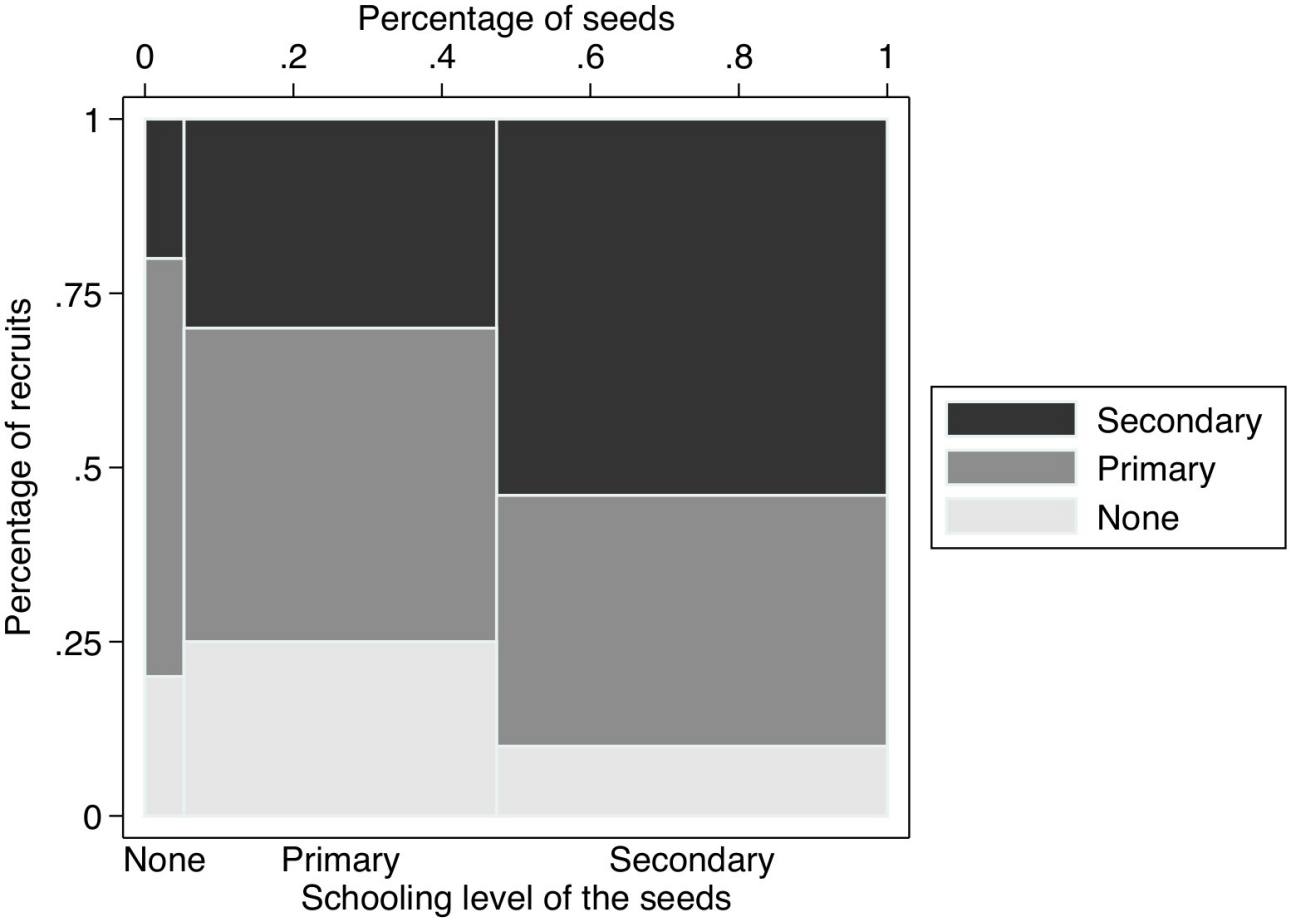
Education attainment by HIV status of the seeds.

In general, recruits were significantly younger than seeds (Fig 2). There were only 13 recruits that were older than the seed who recruited them (13.7%), whereas there were 38 recruits (40.0%) who were younger than the seed who recruited them by more than 10 years. Some seeds recruited peers whose age showed large variation. For example, one seed that was 40 years old recruited peers whose age ranged from 18 to 42 years old. Another seed that was 53 years old recruited peers whose age ranged from 29 to 59 years old. Sixteen recruits (16.8%) had never been to school, 39 had attended primary school (41.1%) and 40 (42.1%) had attended secondary school. Forty-six out of 95 recruits (48.4%) had a schooling level that was similar to the seed that recruited them, whereas 16 out of 95 recruits had a higher schooling level than the seed who recruited them (16.8%) and 33 out of 95 recruits (34.8%) had a lower educational level than the seed who recruited them (Fig 3).

### Relationship between seeds and recruits

**Fig 4:**
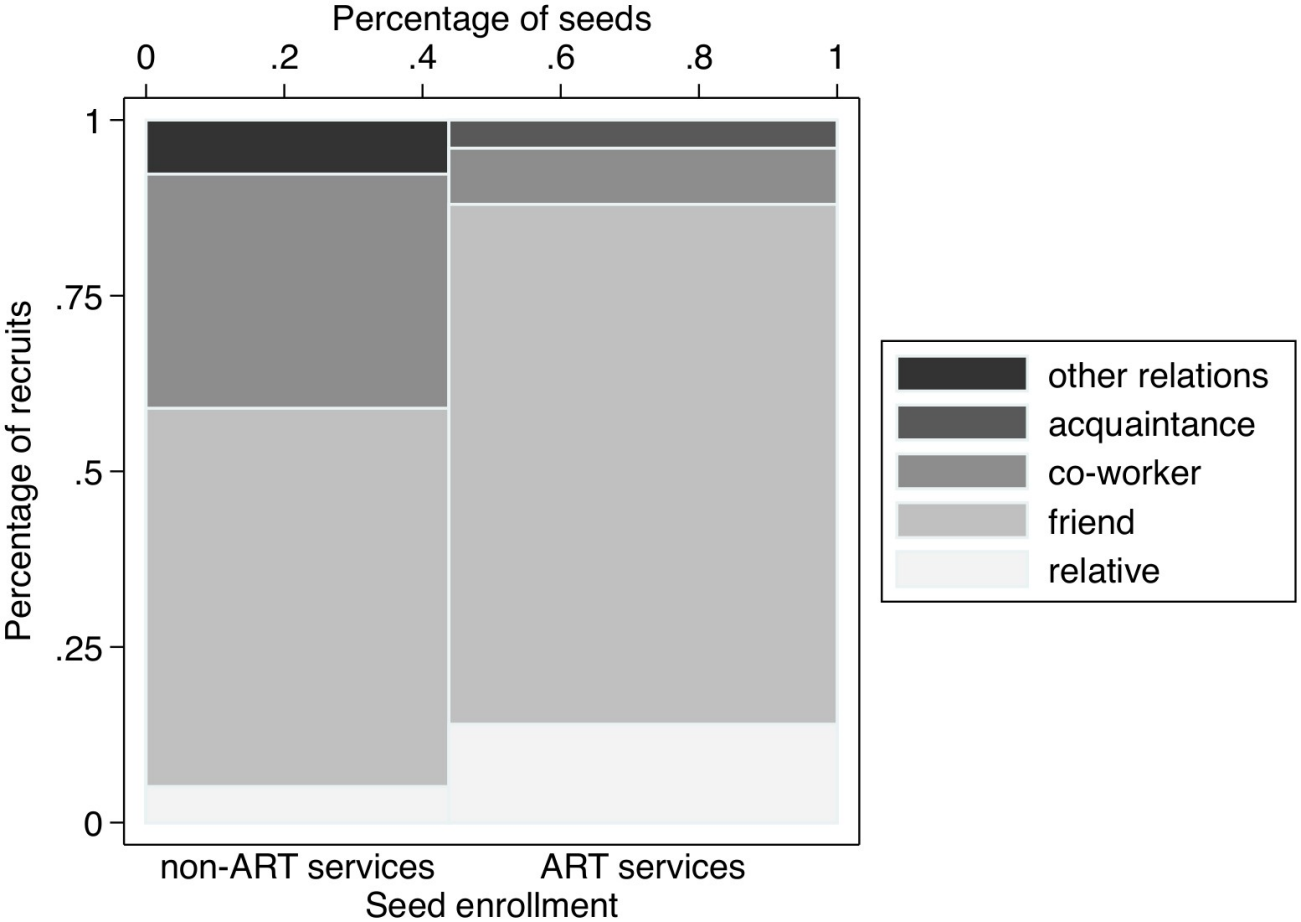
Seed to recruit relationship by the HIV status of the seeds.

Most recruits were approached by a seed they considered a friend (58/89, 65.2%), whereas 9/89 recruits were approached by a seed who was relative (10.1%) and 17/89 were approached by a seed who was a co-worker (19.1%). The relation between recruits and the seed who approached them varied significantly according to the seed’s engagement in HIV care and treatment: seeds who were users of ART services were more likely to recruit peers who were friends or relatives, whereas seeds who were users of other services or who were community members were more likely to recruit co-workers (Fig 4).

### HIV self-testing experience of recruits

Among the 89 recruits who completed the interview, 78 (87.6%) said they immediately self-tested upon receipt of the kit (Table 1), and 81 (92.0%) self-tested at home. Virtually all recruits (87/89, 97.8%) reported that the seed informed them about how to use the HIVST kit, and 80/89 recruits reported reading the instructional leaflet that accompanied the HIVST kit (89.9%). Only one of the recruits (1.1%) reported that it was “somewhat hard” to perform HIVST and 81 out of 88 recruits (92.1%) stated that they trusted the results they obtained through HIVST.

**Table 1:** Utilization of HIVST kits among recruits.

	n/N	%
**Context of HIVST**		
Tested shortly after being offered HIVST kit	78/89	87.6
Used HIVST kit at home	81/88	92.1
Informed by seed about how to use HIVST kit	87/89	97.8
Read instructional leaflet	80/89	89.9
Trust results that were obtained during HIVST	81/88	92.1
Reported that performing HIVST was "not hard at all"	88/89	98.9

### Adverse events, accuracy and yield

In Table 2, we report the main results of the trial of peer-distribution of HIVST kits. There were a very limited number of reported adverse events. None of the recruits who completed the survey reported being coerced to test (Table 1). Two of the seeds reported facing hostile reactions, from potential recruits or their families. According to confirmatory testing, there were 4 HIV-positive recruits (4.2%). Compared to this reference, the sensitivity of HIVST was 100% according to both the recruits’ reading of their own result, and the health worker’s checks of the HIVST kits that were returned. According to the recruits’ own interpretation of HIVST results, the specificity of HIVST was 92.8% vs. 97.8% according to the health workers’ reading of the same HIVST kits.

**Table 2:** Outcomes of HIVST distribution trial.

	n/N	%
**Adverse events**		
Seed faced hostile reaction	2/19	10.5
Recruit forced to test/coerced	0/89	--
**HIVST accuracy**		
HIVST results read by counselor		
Sensitivity^1^	4/4	100.0
Specificity^2^	88/90	97.8
HIVST results reported by recruit		
Sensitivity^1^	4/4	100.0
Specificity^2^	77/83	92.8
**Prior HIV testing history of recruits**		
Never tested	23/89	25.8
Tested more than a year ago	42/89	47.2
Tested within last year	24/89	27.0
**HIV diagnosis at time of HIVST**		
Previously diagnosed	1/87	1.1
Learned HIV-positive	3/87	3.5
Received false positive	6/87	6.9
Learned HIV-negative	20/87	23.0
Repeat HIV-negative test	57/87	65.5

Notes:

^1^ sensitivity refers to the proportion of HIVST results interpreted as positive, when the confirmatory test was itself positive;

^2^ specificity refers to the proportion of HIVST results interpreted as negative, when the confirmatory test was itself negative.

The sample size varies across outcomes of interest because 1) some outcomes were calculated for seeds, whereas others were calculated for recruits; 2) there were some non-responses on various questions during the recruits’ interviews.

**Fig 5:**
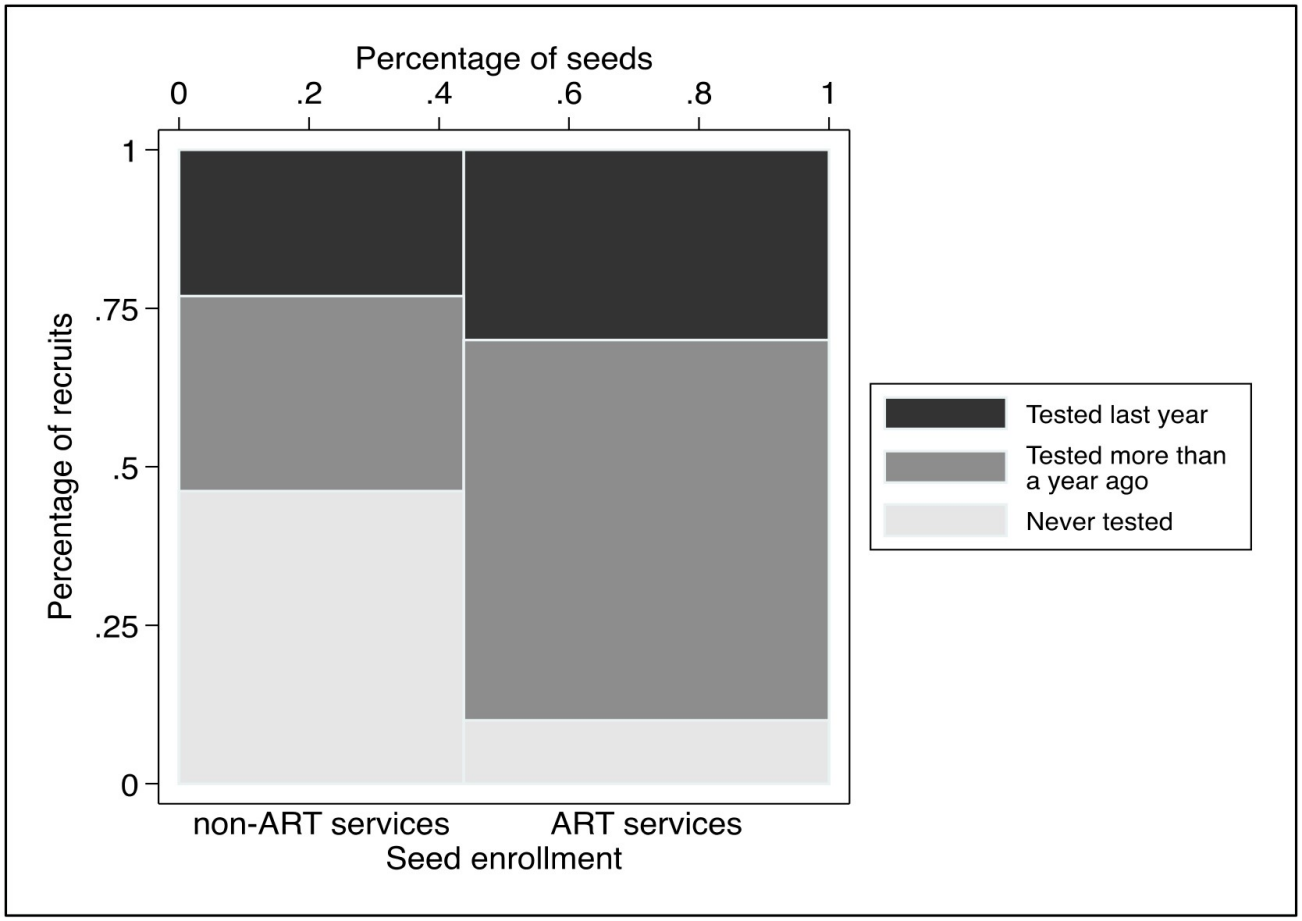
Peer-distributed HIVST outcomes, by mode of enrollment of the seed. Notes: The graph represents the distribution of recruits by mode of enrollment of the seed on the x-axis, and the distribution of recruits by HIVST outcome on the y-axis. The differences in outcomes between recruits from seeds enrolled at ART services vs. non ART services were significant at the p<0.05.

Among recruits, 23 out of 89 reported never having been tested (25.8%) whereas 42 out of 89 had last tested for HIV more than a year ago (47.2%). The HIV testing history of recruits differed significantly between those approached by seeds that were ART users and other seeds. In particular, a much larger proportion of the recruits of non-ART users and community members had never been tested (Fig 5). One recruit had already been diagnosed (1.1%) whereas 3 recruits learned that they were HIV-positive through HIVST (3.5%). Most recruits either learned that they were HIV-negative for the first time (20/87, 23.0%) or received a repeat HIV-negative test (57/87, 65.5%).

In total, 84/89 recruits reported being satisfied with their HIVST experience (94.4%), and 85 out of 88 (96.6%) reported that they would recommend using HIVST to friends and relatives. During the one-month follow-up, all the 19 seeds said they would recommend self-testing to others, and also said they would definitely be ready to continue with kit distribution to their friends who may need them.

## Discussion

Women have primarily served as distributors in prior trials of the secondary distribution of HIVST kits, but our trial in Uganda showed that men could also fulfill that role. The distribution of HIVST kits through the peer networks of men had high uptake. More importantly, this peer model of distributing HIV self-test kits did not seem to increase incidence of coerced testing replicating findings from other HIVST studies [7, 21, 22]. It also reached men who had not previously tested for HIV showing potential to contribute to the first 90% of the UNAIDS targets which aims to diagnose 90% of all people living with HIV. We thus found one undiagnosed HIV case for every 6.3 seeds enrolled.

In this study, we strengthened the measurement of HIVST outcomes in trials of the secondary distribution of HIVST, by enrolling recruits into the study, and retrieving their used HIVST kits. This allowed directly assessing HIVST outcomes, whereas prior trials [14] have relied solely on indirect reports from seeds to assess the experience of HIVST recruits. We also exploited the stability of the results of OraQuick test results, even in sub-optimal storage conditions, to conduct quality control on HIVST [19].

Our study has several important limitations. First, it did not include a control group. As a result, we cannot conclusively argue that the peer-based distribution of HIVST kits helps improve HIV diagnosis and case finding. Second, even though all newly diagnosed MLWH were linked to clinical services following HIVST, we did not assess their initiation of ART. Third, our estimates of the uptake of peer-distributed HIVST may be inaccurate if seeds misreported the number of individuals they offered an HIVST kit to. If they under-estimated that number, then the uptake of peer-distributed HIVST may be lower than we estimated in this study. Fourth, HIVST produced several false positives, i.e., recruits who interpreted their HIVST results as “positive”, but later received a negative confirmatory test result. In most of these instances, recruits misinterpreted a faint grey line that appeared in the “test” location of the test kits. Fifth, some recruits may have been prompted to participate in the study to obtain the monetary contribution offered to cover transportation costs. In settings where such incentives are not available, the uptake of HIVST following peer distribution may be lower. Finally, there is very limited generalizability from our study given the small sample size.

Our study nonetheless suggests that the secondary distribution of HIVST kits through peer networks of men may help reduce the gender gap in HIV diagnosis in African countries [5]. It may be particularly useful in addressing the HIV epidemics affecting fishing communities of the great lakes of eastern Africa, where men are at high risk of HIV infection and HIV testing services are limited [18]. This model should be tested in future randomized trials at the individual and facility levels, with linkage to care and viral suppression as key outcomes.

In summary, we found a peer model of distributing HIV self-test kits to men in fishing communities highly acceptable with no serious adverse events to either the distributor (seeds) or the user (recruits). The recruits were able to correctly perform their own HIV self-testing following training from their peer. Despite the model being highly acceptable with high clinic attendance for confirmatory testing HIV treatment initiation was not assessed. Larger randomized trials are warranted to investigate these outcomes more rigorously.

## Supporting information

S1 ScriptEng Script in English used in training the seeds in the distribution of HIV self-test kits to their peers (recruits). (DOCX)

S1 ScriptRun Script in Runyoro (the local language) used in training the seeds in the distribution of HIV self-test kits to their peers (recruits). (DOCX)
