# Supplementary material for: A pilot trial of the peer-based distribution of HIV self-test kits among fishermen in Bulisa, Uganda
Source: PLoS One. 2018 Nov 29;13(11):e0208191. doi: 10.1371/journal.pone.0208191 (PMC6264512; doi:10.1371/journal.pone.0208191)
Supplement: S1 ScriptEng — (DOCX) [file pone.0208191.s001.docx]

**Seeds procedure document**

**HIV self-testing study in Buliisa, Uganda**

**Background**

HIV is a virus which causes AIDS and destroys the body’s immune system thus making the body susceptible to diseases. HIV is mainly spread through sexual intercourse, sharing sharp objects with those infected, mother to child transmission and blood transfusion. Africa the largest number of people living with HIV hence our interest in finding effective ways of finding people who are living with the virus and are not yet identified.

**What is HIV self-testing (HIVST)?**

Self-testing is a process through which an individual collects a sample, conducts and interprets a test on his/her own. Here we are concentrating on an oral test called OraQuick manufactured by OraSure Technologies in the United States of America.


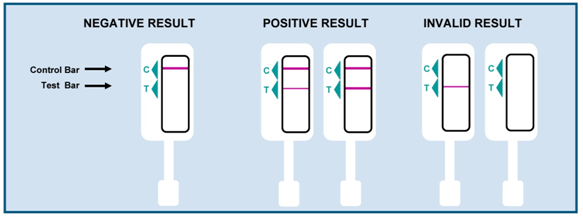
Many studies in Africa and other parts of the world have found this test kit to be highly accurate when compared with blood testing. This means that the results obtained are similar to results obtained through testing at the health facility [blood]. Line at C it means negative; two lines on both C and T means positive; no line or line at T only means invalid [see diagram below]. Remember the test has a window period during which HIV antibodies cannot be detected by the test. Therefore, all negative tests should be confirmed within three months after the first test. Red fluid will rise along the test device and pass the T and C indicators --- this happens in all cases and does not mean that the test will be positive.

Please **always** use the cotton wool buds for demonstrating to the recruits **each** time you are distributing the test kit. If you don’t do this, it is highly likely that the recruit will not do the test correctly thus may affect the results that they may see.

**Benefits and risks of HIVST**

There are no direct benefits associated with participation in the study. The information we will collect during the study may help develop new ways to prevent HIV transmission in this population and in other similar settings in Uganda.

The risks associated with participation in this study consist primarily of a possible breach in confidentiality. By this we mean that, during the course of the study, some people may come to find out about the answers you have provided to some of our questions. We will put in place a number of safeguards to avoid this, but we cannot guarantee that this will not happen. If you feel that you have been harmed during your participation in the study, you should report this to the study team, who may be able to help.

All recruits are asked to go to the health facility for a visit and interview. A small sum (20,000 Ugandan Shillings) will be given to offset the costs of transportation as well as lost wages you may have incurred on that day.

**Peer distribution**

Who is a seed? A fisherman or a fish trader within the three landing sites of Buliisa district. He was identified through access to health facility or through village health teams and is tasked with distribution of HIV self-test kits to a recruit [fellow fisherman or fish trader].

Accepting an offer of an HIVST kit by the recruit is **voluntary** and refusing to accept is not punishable by anybody and will not affect receipt of any health services. Neither family members nor friends or anyone else should be coerced to self-test.

The test result may turn out negative, positive or invalid. Ensure that recruits understand what to do in case of any of the three outcomes as described during the training of seeds. In general, the HIVST kit and the accompanying coupon should ideally be returned to the named person at the health facility. All positive tests will be confirmed through the existing blood-based method followed by early initiation of ART. For this research, negative and invalid tests will also be confirmed at the health facility.

Peer distribution has proved a significant method of reaching out to many people through a network of peers. It was chosen here because we want to reach out to fishermen and fish traders who are normally busy, find it hard to reach the facility due to costs and distance.

**Characteristics of the recruits**

Ask your peer the following questions to check their eligibility to participate in this study:-

1. Are you a fisher or do you trade in fish? *[Eligible only if either fisher man or trades in fish].*
2. What is your age? *[Eligible if 18 years or older; if <18 refer to health facility]*
3. When was the last time you had an HIV test? *[Eligible if never tested before, tested within last year or more]*

***Note: person eligible if they meet all the three criteria above. Family members including spouses are not eligible.***

**Managing positive tests and potential adverse events**

Use the counselling knowledge to assist recruits who are struggling to accept a new HIV positive diagnosis. Kindly call your counsellor [Aisha or Juliet] to involve them with further counselling and linkage into care. Juliet or Aisha will be able to offer on spot phone counselling and psycho-social support. Please ensure the recruit that such disclosure will remain extremely confidential and you are not supposed to discuss this with anyone in your household or community.

Seeds **must** report *any* incidence of adverse events [physical violence, psychological violence, suicide or suicidal tendencies, homicide or homicidal tendencies, any other events] to Aisha and Juliet immediately. Please do not restrict the recruits on what they can and they cannot report. Inform recruits that they can report coercion to Aisha or Juliet.
