# Supplementary material for: A pilot trial of the peer-based distribution of HIV self-test kits among fishermen in Bulisa, Uganda
Source: PLoS One. 2018 Nov 29;13(11):e0208191. doi: 10.1371/journal.pone.0208191 (PMC6264512; doi:10.1371/journal.pone.0208191)
Supplement: S1 ScriptRun — (DOCX) [file pone.0208191.s002.docx]

**Akatabu akaroho endagiro yokugabira abanyani ebyokwekebera wenka silimu.**

**Okutolereza ha kwekebera wenka silimu omu Buliisa, Uganda**

**Background /Endugiro**

HIV niko akahuka akaleta omunywerero nukwo obusirikale obulinda omubiri nibuhwa amani omuntu natandika kurwararwara. Kanu akahuka ka HIV kakira muno kutulirwa nikaraba mukuterana omukazi nomusaija batajwaire akapira/condom, kukoleseza hamu ebintu ebikucumita byoona, mama kutulira omwanawe hamu nokugabirwa esagama. Africa niyo ekusingira kimu abantu nyamwingi abaina akahuka komunywerero omunsi yoona,nahabwekyo tukusikirizibwa kutolereza emihanda nukyo abantu abatemanyire nti bakakwatwa ira akahuka ka silimu basobole kwemanya.

**Kwekebera wenka nikyo kiki (HIVST)?**

Kwekebera wenka nukwo omuntu kweihaho wenka ebyokukebera,akakebera kandi akasoma ebirugiremu wenka. Hanu tugumiire ha kukebera kwo munwa nitukolesa OraQuick ekozerwe OraSure Technologies mu United States of America.

Okutolereza mu Africa nebicweka ebindi ebyomunsi bakakizora nti ekyokukebera kinu kwo mali kikwoleka amazima obwo nibakilengesaniza nokukebera esagama. Kinu nikimanyisa nti rizaitu ezikurugamu zikusisana nezo ezibakukebera omwirwaro nibakozesa esagama . omusitale gumu ha” C”gumanyisa toina akahuka(negative); emisitale ebiri ha “C” na”T”kimanyisa oina akahuka ( positive); busaho musitale gwona rundi haloho gumu ha “T”ebyo bifire( invalid) [rora ebisisanihansi]. Ijuka haloho akahuka obukaba keserekere kandi hanyuma yokukakebera nikanga kuzoka.nahabwekyo, abantu boona abasanga bataina akahuka kasilimu hakwekebera okukubanza basemera bagaruke omurwaro hanyuma yemyezi esatu nukyo bagumye rizaitu zabu.Amaizi gakutukura gaija kutemba gahike ha “T” na” C” omukutandika kwekebera baitu ekyokibaho kandi oteralikira okatekereza nti oli murwaire.


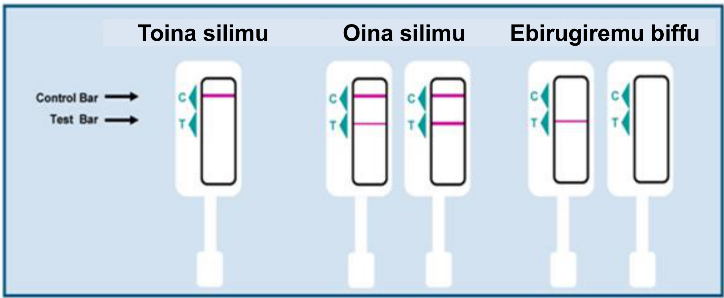


Bairaba kozesaga obupamba bwomumatu bulibworabaga noyegesa ogu wena oworaba nohereza ebwokwekebera wenka (HIVST) kit.obworaba otakikozere kikusora kurugiramu abo aboragabira butasoma kurungi nukyo baketegereza ebirugiremu omukwekebera

**Omugaso nobunkenke ebiri omukwekebera wenka.**

Tiharumu magoba gamani iwe nko muntu gokufuna mukweteraniza omu kutolereza kunu. Amakuru agaturatunga kuruga omukutolereza kunu gasobora kuyamba abantu baitu kufuna enkora endi empyaka eyokutangira kutura akahuka ka silimu no muihanga lyoona.

Obunkenke obutukunihira kurugira omukweteraniza kwawe omukutolereza kunu kuli abantu bandi kukikenga nti oli hamu naitwe,tukumanyisa nti abantu abandi nibasobora kumanya engarukwamu yawe ha bihabuzo byaitu.itwe twija kurora nti twehazire ekyo kubaho,baitu titukugumya nti tibamanye. Obworaba nohura nti okweteraniza omukutolereza kunu kukuleteire oburumi,gambira abakuru omukutolereza kunu, nukwo bakuyambe.

Abo boona abamuraha ebyokwekebera binu baina kubunga hairwaro bahabuzibwe ebihabuzo bimu. Akasente kataito(20,000 Ugandan Shillings) kabayambe habwobwire hamu na transport nebyoebimwerekereize habwokwija hairwaro.

**Kuherezebwa ebyokwekebera kuruga omubanywani.**

Oha Agaba ebyokwekebera wenka”seed”? omusohi rundi omusubuzi wensumaki kuruga ha myaro esatu eya Buliisa district.Ateirwemu obwesigwa eirwaro rundi VHT agabege obyooma bwokwekebera wenka nukwo agabire asohi hamu nabasubuzi bensamaki.

Okwikiriza kugabirwa akooma kokwekebera wenka tikikuhambirizibwakandikwanga tikuleta kufubirwa hamu nokuhakanwa kufunaga obujanjabi obundi.Busaho eka yoona rundi omuntu mweka egyo aina kuhambirizibwa kukolesa akooma kokwekebere wenka.

Ebirugire omukwekebera wenka bisora kuba ataina kahuka komunywerero, rundi karumu, rundi bifiire. Junanizibwa kurora nti abohaire akooma kokwekebera boonka betegereize ebirugamu ebikusoboora kurugamu ebisatu.Kutwaliza hamu, akooma kokwekebera wenka, hamu nebaruha endi (referal coupon) bina kugarurwa hairwaro omuntu ogu ayabikwasibwe.

Abantu boona abarasanga baina akahuka ka siliimu baija kugarukamu bakeberwe ahairwaro omusahi kandi batandike omubazi gwa ARVS nibukyali.Ha kutolereza kunu, abantu abaraba bataina nabo abaraba okukebera bifire(invalid) nabo baija kugarukamu bakeberwe omusahi ahairwaro.

Okugabirangana omubanywani bakaguzora kuba muhanda gwomugaso muno kuhikiira abanywani baingi. Bakakomamu omulingo gunu habwokuba kikwanguha kuhikira abasohi abataina bwiire habwokuba kibagumiira kugenda hairwaro hamu nokusasura ebisale bya transport.

**Ekika kyabanywani bawe abatukutooza**

Habuza omunywani wawe ebihabuzo biinu nukwo oramanya obyaraba ahikire kweteraniza omukutolereza kunu.

1. Osoha? Rundi osubura ensamaki? *[muhe akooma obwaraba asoha rundi asubura ensamaki].*
2. Oina emyaka eingaha? *[muhe akooma obwaraba aina emyaka 18. Obwaraba atakahikize emyaka 18 musindike hairwaro]*
3. Okasembayo dihi okwekebeza? *[muhe akooma obwaraba atakekeberaga rundi yayekebeire omwaka ogwahoire rundi ira muno)*

***Kinu kikuru:abantu abakuhikiriza orupimyo orwaharuguru(3) nibo bonka abaina kuhebwa obyooma byokwekebera.abantu aba haka hamu nabagonzebwa banyu tibaina kuherezebwa obyoma kwekebera bonka.***

**Okurolera abanywani abarasanga baina akahuka komunywere hamu nebigwa bitaraganize.**

Kozesa obukugu oyambe abanywani abakutalibanizibwa kwikiriza ebirugire omukwekebera akahuka komunywerero basanga karumu.Bambi yeta kansala [Aisha rundi Juliet] nukwo bababudebude kandi babasindike omwirwaro bafune obujanjabi obukuhonderaho. Juliet hamu Aisha baija kubabudabuda aho naho hasimu kandi bongere kubagumya. Bambi omunyani wawe mugumize ntingu ebyakugambiire toija kubibaza omubantu abandi boona kandi otabinyumiza abomunju yawe na bantu abomukicweka ekyo.

Abagaba obyooma baina kuleta repota hali ebigwa bitaraganize gamba nka [okurwana, okubihizangana, okweita rundi kutinisiriza kweita,kwiita abahaka rundi kutinisiriza kubaita, nebindi byona] owa Aisha hamu na Juliet ahonaho. Bairaba mutakangisiriza abamuhaire obyooma ebibaina kubaza nebibataina kubaza.Abanyani banyu mubagambe nti baina obugabe kugambira Aisha hamu na Juliet obubaraba bahambisirizibwe kwekebera.
